# Physiology of pericardial fluid production and drainage

**DOI:** 10.3389/fphys.2015.00062

**Published:** 2015-03-18

**Authors:** Konstantinos Vogiatzidis, Sotirios G. Zarogiannis, Isaac Aidonidis, Evgeniy I. Solenov, Paschalis-Adam Molyvdas, Konstantinos I. Gourgoulianis, Chrissi Hatzoglou

**Affiliations:** ^1^Department of Physiology, Faculty of Medicine, School of Health Sciences, University of ThessalyLarissa, Greece; ^2^Laboratory of Physiological Genetics, Institute of Cytology and Cytogenetics, Siberian Branch of the Russian Academy of SciencesNovosibirsk, Russia; ^3^Department of Respiratory Medicine, Faculty of Medicine, School of Health Sciences, University of ThessalyLarissa, Greece

**Keywords:** mesothelium, pericardiac fluid turnover, pericardium, serosal membranes, transmembrane transport

## Abstract

The pericardium is one of the serosal cavities of the mammals. It consists of two anatomical structures closely connected, an external sac of fibrous connective tissue, that is called fibrous pericardium and an internal that is called serous pericardium coating the internal surface of the fibrous pericardium (parietal layer) and the heart (visceral layer) forming the pericardial space. Between these two layers a small amount of fluid exists that is called pericardial fluid. The pericardial fluid is a product of ultrafiltration and is considered to be drained by lymphatic capillary bed mainly. Under normal conditions it provides lubrication during heart beating while the mesothelial cells that line the membrane may also have a role in the absorption of the pericardial fluid along with the pericardial lymphatics. Here, we provide a review of the the current literature regarding the physiology of the pericardial space and the regulation of pericardial fluid turnover and highlight the areas that need to be further investigated.

## Introduction

The pericardium is one of the serosal cavities of mammals (Michailova and Usunoff, 2006). It is a fibrous—serosal conical sac enclosing the roots of the aorta and the pulmonary artery (Chinchoy, 2005). In humans, pericardium is located inside the middle mediastinum posteriorly to the sternum and the cartilages of the third to seventh left rib. Normally, it is not in contact with the frontal wall of the thoracic cavity (Frick et al., 1985; Chinchoy, 2005). Laterally, it is held together with the mediastinal parietal pleura. Pericardium isolates the heart from the adjacent tissues, allowing it's free movement within the boundaries of the pericardial cavity and is filled with a small amount of fluid which is called pericardial fluid (Chinchoy, 2005).

## Anatomy and histology

The pericardium consists of an external sac of fibrous connective tissue, called fibrous pericardium and an internal called serous pericardium. The latter coats the internal surface of the fibrous pericardium and the heart. Arterial branches from thoracic aorta, right and left pericardiophrenic artery (internal mammary artery branches), are responsible for the blood supply of the whole pericardium while the venous drainage is accomplished through the venae pericardiales which drain to the azygos vein, to the superior vena cava or to the brachiocephalic (Chinchoy, 2005). The pericardium is innervated by the two phrenic nerves, each one giving an afferent branch (pericardial branch) (Randall and Ardell, 1985; Ardell and Randall, 1986; Chiou et al., 1997).

The thickness of the pericardium increases proportionally to the size of the heart and the pericardial cavity, with the exception of humans who have considerably thicker pericardium compared to the mammals with the same heart size (human 1–3.5 mm, sheep 0.32 ± 0.01 mm, pig 0.20 ± 0.01 mm) (D'Avila, 2003). The serous pericardial membrane covers the outside of the heart (visceral pericardium), extending a short distance beyond the atria and ventricles on the great vessels and lines the inside of the fibrous sac (parietal pericardium). The visceral pericardium is also refered to as epicardium and is in continuance with the parietal pericardium (Chinchoy, 2005; Jöbsis et al., 2007).

The parietal lamina of the serous pericardium is composed of a monolayer of flattened, squamous-like, mesothelial cells. Mesothelial cells rest on a thin basement membrane supported by connective tissue stroma in a narrow submesothelial space. The connective tissue stroma contains variously oriented layers of collagen fibrils and small elastic fibers (Ishihara et al., 1980; Mutsaers, 2002). The luminal surface of the mesothelial cells has well developed microvillous border with occasional cilia. The latter bear friction and increase the surface area for fluid transport. There are junctional complexes between adjacent mesothelial cells that consist of desmosomes, which reinforce intercellular adhesion and zonulae occludentes. All of these morphological characteristics form permeability barriers. Actin-like filaments are present in microvilli and in immediate subjacent regions of the cells. These filaments mediate changes on cell shape. Intermediate filaments are associated with desmosomes and form bundles in the perinuclear regions, which provide structural support to the cytoplasm (Ishihara et al., 1980).

Between the mesothelial cells of the parietal pericardium, not the epicardium, there are milky spots, similar to those in the omentum and mediastinal pleura, bulging toward the pericardial cavity. These structures are enclosed with cuboidal mesothelial cells (Takada et al., 1991; Mutsaers, 2002; Michailova and Usunoff, 2006). These openings provide direct access to the underlying submesothelial lymphatic system allowing rapid removal of fluid and cells from the pericardial cavity (Takada et al., 1991). Inside the pericardial cavity and submesothelial layers of the pericardium, there are resident macrophages, readily available in case of immunological response (Ishihara et al., 1980; Mutsaers, 2002; Michailova and Usunoff, 2006).

Histological studies on parietal pericardium of rodents have shown the presence of circular fenestrations (“pores”; diameter up to 50 μm), which connect the pericardial cavity with the adjacent mediastinal pleural cavities, except the positions that are held by the adipose tissue (Nakatani et al., 1988; Mohrman and Heller, 2006). Both their stomata and lumen are covered with mesothelial cells and below them elastin and collagen fibers (Fukuo et al., 1988; Nakatani et al., 1988). The functional significance of the pericardial “pores” remains unclear, however labeled erythrocytes have shown to pass through them from one cavity to the other (Fukuo et al., 1988).

The epicardium has common morphological features with the parietal pericardium but there are some functional differences. On the laminal surface there is a monolayer of mesothelial cells, lying on a thin basal membrane (Mutsaers, 2002; Jöbsis et al., 2007). The proportion of cuboidal cells is greater in the epicardium than in the parietal pericardium (Michailova and Usunoff, 2006). Underneath the basal membrane there is a dense network of collagen and elastic fibers, full of hydroxyproline, which embryologically stems from extracardiac tissue and doesn't infiltrate the underlying myocardium. The fibers are parallel to each other, in multiple layers and crossing the heart surface from diagonally to vertically, compared to the myocardial cells (Simionescu et al., 1993; Jöbsis et al., 2007). These mechanical properties play a role in the residual stress and passive stiffness of the heart (Przyklenk et al., 1987; Jöbsis et al., 2007).

## Pericardial fluid

The composition of the normal human pericardial fluid is difficult to define. All available data have been obtained either by cardiothoracic surgery patients or from animals. This probably compromises the data validity (Ben-Horin et al., 2005). However, the pericardial fluid is a plasma ultrafiltrate having specific characteristics just like the pleura fluid (Mauer et al., 1940; Holt, 1970). Volumetric studies have shown that the pericardial fluid volume is directly analogous to the animal size: in rabbits 0.4–1.9 mL, in dogs 0.5–2.5 mL and in adult humans about 20–60 mL (average 15–35 mL) (Vesely and Cahill, 1986; Ben-Horin et al., 2005).

Pericardial fluid coloring studies report that the fluid distribution inside the cavity is heterogeneous. The largest amount is inside the atrioventricular and the intraventricular sulcus, the superior and the transversal sinus, especially on the supine position (D'Avila, 2003). Nevertheless, there are some pharmacokinetic studies that show that the pericardial fluid is stirring up constantly and thus the supplement's composition is the same regardless the position (Chinchoy, 2005).

Regarding the cell population, studies in human normal pericardial fluid have shown the presence of a heterogenous cell population. There are mesothelial cells, lymphocytes (53%), glanulocytes (31%), macrophages (12%), eosinophils (1.7%), and basophils (1.2%). This means that the pericardial fluid “lymphocytosis” should always be under critical consideration and characterized as pathological only when it exceeds 60% of the whole cell population (Gibson and Segal, 1978a; Benhaiem-Sigaux et al., 1985).

The pericardial fluid is considered to be a plasma ultrafiltration product, like other serosal cavity fluids. Studies in greyhounds showed that the concentrations (in mmole kgr H_2_O^−1^) of Na^+^ (150.5 ± 0.72), Cl^−^ (123.2 ± 0.71), Ca^2+^ (1.92 ± 0.04), and Mg^2+^ (0.85 ± 0.09) were lower in the pericardial fluid than in the plasma. On the contrary, the concentration of K^+^ (3.81 ± 0.07) was higher than the plasma, which was attributed to the K^+^ leakage from the myocardial interstitium toward the pericardial cavity, during systole (Holt, 1970; Gibson and Segal, 1978b). The protein concentration was also lower with different proportion of protein fractions; from higher to lower concentration being albumin, globulins, macroglobulins, and fibrinogen. Finally, the pericardial fluid osmomolarity was lower than the plasma (Gibson and Segal, 1978b). Given the net filtration gradients of the substances above, it is obvious that the normal pericardial fluid is a transudate (Holt, 1970; Gibson and Segal, 1978b). However the relatively high protein and LDH concentrations raise caution as far as the applicability of Light's criteria is concerned (Gibson and Segal, 1978b; Meyers et al., 1997; Burgess et al., 2002; Ben-Horin et al., 2005).

## Physiology

The normal pericardium contributes in important functions. In is necessary for: (1) lubricating the moving surfaces of the heart, (2) stabilizing the heart anatomic position, (3) isolating the heart from the adjacent anatomical structures, prohibiting the adhesion formation, the inflammatory or neoplastic extention, (4) limiting heart dilatation during diastole, reducing the endomyocardial tension, (5) preventing cardiac hypertrophy in pressure overload conditions, (6) reducing the right ventricular impulse work in left ventricular overload conditions, (7) the ventriculoatrial blood retrogression prevention during high end-diastolic ventricular pressures, (8) the preservation of the negative endothoracic pressure, which is crucial for the atria blood filling, (9) the nervous stimulation response and regulation of the cardiac frequency and arterial blood pressure, (10) the formation of a hydrostatic compensation system ensuring that end-diastolic pressure remains the same at all hydrostatic levels and the Frank–Starling mechanism is functional (Holt, 1970; Goto and LeWinter, 1990; Cinca and Rodriguez-Sinovas, 2000).

Many studies in both parietal and visceral pericardium (human, canine, pig) have shown the same mechanical properties, only with quantitative differences. *In vitro* studies in canine parietal pericardium demonstrated the presence of viscoelastic response, mainly attributed to the presence and the arrangement of the elastic and collagen fibers (Lee and Boughner, 1981, 1985). These parietal pericardium properties are responsible for (1) its participation to ventricular volume modulation, (2) the intraventricular interaction, and (3) the participation to the ventricular diastolic pressure/volume relatioships (Lee and Boughner, 1981; Maruyama et al., 1982; Takata et al., 1997; Gibbons Kroeker et al., 2003).

*In vitro* epicardial studies (humans, pigs), proved the presence of elastic properties that have been attributed to the composition and orientation of the connective tissue. Specifically, the human epicardium undergoes an oblong and circumferential systolic shortening, therebe the energy gathering during the diastolic period may contribute to the passive mechanical properties of the myocardium (Lorenz et al., 2000; Jöbsis et al., 2007). In this way, the epicardium seems to be participating in the ventricular end-diastolic volume control (Jöbsis et al., 2007).

## Hydrodynamics

Under normal conditions, the human pericardial cavity contains 20–60 ml of fluid (Chinchoy, 2005). Moreover, it has been determined that the whole amount of pericardial fluid drains through the lymphatic capillary bed every 5–7 h, in sheep (Yuan et al., 2000). The pericardial fluid volume is determined by the equilibrium between production and drainage. There is strong evidence that the pericardial fluid is derived by plasma ultrafiltration through the epicardial capillaries (and probably the parietal's pericardium), as well as a small amount of interstitial fluid from the underlying myocardium, during the cardiac circle (Stewart et al., 1997). The fluid drainage is mainly accomplished through the parietal pericardium lymphatic capillary bed (Yuan et al., 2000). Nevertheless, the whole procedure is not fully elucidated because of the difficulty to study pericardial fluid dynamics under normal conditions (Shabetai, 2003).

**Figure 1:**
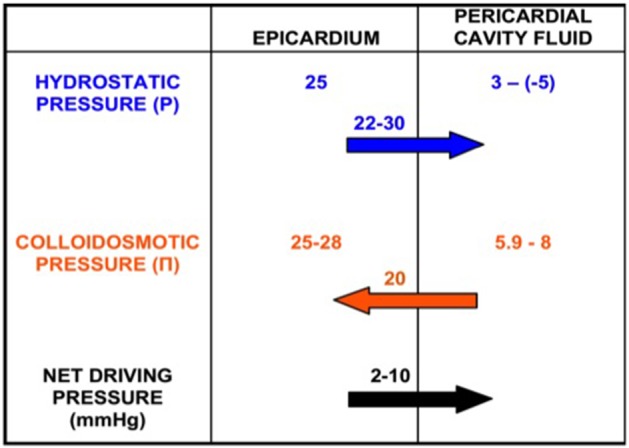
**Production of pericardial fluid as a net pressure phenomenon**. Epicardial and pericardial cavity hydrostatic (P) and colloidosmotic (Π) pressures. There is a net pressure difference of 2–10 mmHg that drives the fluid from the epicardium toward the pericardial cavity.

The fluid movement through the pericardial laminae is a hydrostatic/osmotic pressure equilibrium, between the microvasculature and the cavity (Yuan et al., 2000). As shown in Figure 1, according to the Starling equilibrium:

(1) $$\text{Fluid Movement}=\text{L}\times \text{S}[({\text{P}}_{\text{cap}}-{\text{P}}_{\text{p}})-\sigma \times (\Pi_{\text{cap}}-\Pi_{\text{p}})]$$

P and Π stand for hydrostatic and osmotic pressures inside the capillaries (cap) and pericardial cavity (p), respectively. L is a membrane conductance constant for liquids, S is the surface area and σ is the osmotic constant of the endothelium/interstitial tissue barrier for proteins (Yuan et al., 2000).

The P_p_ measurement is challenging. The main problems for the direct measurement of the P_p_ were the small cavity size (<0.34 mm) as well as the sac deformation that provoved by the catheter insertion. The P_p_ depends on the pericardial fluid volume and accumulation velocity, the cardiac and respiration circle phase and the measurement position. The large amount and the fluid rapid accumulation results in rapid and great increase in P_p_. This is not present during the gradual fluid accumulation, which allows greater ammounts of fluid without hemodynamic instability. This phenomenon is due to the viscoelastic properties of the parietal pericardium (Holt, 1970). During the diastole (laminae approach) the P_p_ increases and during the systole it decreases (Smiseth et al., 1985; DeVries et al., 2001; Hamilton et al., 2004). The P_p_ becomes more negative during inspiration and less during expiration (in humans ~ 3 mmHg endexpiration and −5 mmHg endinspiration) (Kenner and Wood, 1966; Spodick, 1997). Finally, the P_p_ is about 20 mmHg above the free ventricular wall and almost zero inside the great sulcus (atrioventricular, intraventricular), during the enddiastole (Traboulsi et al., 1992). This ensures the constant fluid movement and homogenous composition, regardless the gathering position (Santamore et al., 1990). The pericardial capillaries stem from the systematic circulation. The venous drainage is accomplished through the superior vena cava (high pressure system). Thus, the P_cap_ is about 25 mmHg, the Π_cap_ is about 25–28 mmHg (Mohrman and Heller, 2006) and the Π_p_ is calculated near 5.9–8 mmHg by using the van't Hoff equation (Ben-Horin et al., 2005).

## Pericardial lymph drainage

The lympatics have high absorbing capacity due to the smooth myocytes, placed circumferentially around the lumen that transforms them into a pump. This pump has a diastolic and systolic period that is controlled by the Frank–Starling mechanism, like the cardiac pump, increasing the absorbing capacity under increased “preload” conditions (Yuan et al., 2000; Quick et al., 2007).

The initial studies on the role of lympatic vessels during pericardial fluid drainage were controversial (Hollenberg and Dougherty, 1969; Miller et al., 1988). Finally, the leading role of the lymphatics was established (Boulanger et al., 1999). The thoracic duct ligation decreased the fluid drainage, without completely blocking it. That was attributed to the complicated structure of the lymphatic capillary bed (Eliskova et al., 1995; Boulanger et al., 1999).

*In vivo* studies in sheep support the notion that the fluid drainage through the lympatics increases proportionally to the volume or pressure increase, as much as four times. This property has been related to the effect of external factors on the lymphatics function as well as to the functional alterations due to the neurohormonal stimulation. These characteristics are extremely important under conditions of fluid accumulation like cardiac tamponade (Miserocchi, 1989; Yuan et al., 2000). Also, in rabbits and mice, but not in sheep, there is a proven communication between the pericardial and the pleural cavity, through “pores” (diameter up to 50 μm), enabling fluid leakage toward the pleural cavity in certain conditions (Nakatani et al., 1988; Boulanger et al., 1999).

## Water, electrolyte, and protein transport

The common mesodermal origin and the simplicity of its isolation established the parietal pericardium as a surrogate tissue for pleural mesothelial tissue studies in small animals (Ishihara et al., 1980; Zocchi et al., 1998). Early studies showed data consistent with the passive diffusion of water, Na^+^, Cl^−^ and small molecules (sucrose, mannitol) through “small” pores and later it was shown that there is a passive paracellular diffusion of macromolecules (albumin, dextranes) through “large” pores (either like permanent channels or like transient cystic formations). In both cases the main morphological and functional barrier proved to be the mesothelium. However, by that time active ion trasport through the parietal pericardium was thought to be minimal (Zocchi et al., 1998; Bodega et al., 2000).

However, a later study that examined this aspect showed that the electrical resistance of the rabbit parietal pericardium is measurable and attributed to the mesothelial barrier since it had greater resistance (10.1 ± 0.9 Ω × cm^2^) compared to the underlying connective tissue alone (1 ± 0.2 Ω × cm^2^) (Bodega et al., 2001). This established the mesothelium as the main barrier for molecular transfer. Furthermore, another study shed more light to the active electrolyte transport through the parietal pericardium showing that it is directed from the basal to the apical surface with a net driving pressure of ~3 cmH_2_O, which is 5-fold higher than the one through the pleural lamenae (Tang and Lai-Fook, 2005). In this study it was also shown that the diffusion constant of the rabbit parietal pericardium for albumin (0.26–0.96 × 10^−8^ cm^2^/s), is independent from its concentration. This finding contradicted the previous data regarding rabbit pericardium, pleura and omentum where the reference values were higher and directly proportional to albumin concentration (Parameswaran et al., 1999a,b; Bodega et al., 2002). This discrepancy was attributed to the experimental conditions and the tissue differences. The pericardium contains a larger proportion of collagen fibers compared to the elastic ones and a higher concentration of hyaluronic acid that render it more stiff as well as less permeable than the other serosal membranes (Tang and Lai-Fook, 2005). Moreover, the characteristics above are related to the higher values of hydraulic and electrical resistance of the pericardium and mainly the mesothelial layer (Bodega et al., 2002; Tang and Lai-Fook, 2005). There are *in vitro* data that the hydraulic permeability of the parietal pericardium is independent of the hydrostatic pressure over the range from 6 to 15 cmH_2_O and directly proportional to the membrane thickness, among the species (Fingerote et al., 1980).

Variance among species seems to be the case also in terms of transmesothelial electrical resistance, an index of ion transport. As mentioned above rabbit parietal pericardium had values in the range of 10.1 ± 0.9 Ω × cm^2^, while in sheep these values were nearly double (22.83 ± 0.4 Ω × cm^2^) (Vogiatzidis et al., 2006). Moreover, in the last study the effects of morphine on the pericardium were assessed and it was shown that the electrical resistance of the pericardium is increased by the application of morphine. The same results were found in the pleura and the peritoneum indicating a common opoidergic influence of the ionic transport capacity of the three serosal membranes (Vogiatzidis et al., 2006; Zarogiannis et al., 2007).

## Conclusions

The study of pericardial space physiology is an area with many things to be discovered. The mechanism of pericardial fluid production is straightforward in physiological conditions, however it needs to be identified what is the exact role of the mesothelial cells both in the recycling of the pericardial fluid as well as with respect to the paracrine function that they possess. Another important challenge would be to dissect the exact contribution and magnitude of each mechanism regulating the recycling of the pericardial fluid. Finally, few things are known about the interplay of mesothelial cells and pericardial fluid. These areas will increase our understanding of the physiology of the pericardial space once explored as well as they will provide us with new insights regarding drug development in the context of pericardial effusions.

### Conflict of interest statement

The authors declare that the research was conducted in the absence of any commercial or financial relationships that could be construed as a potential conflict of interest.
